# Tribological and mechanical investigation of multi-directional forged nickel

**DOI:** 10.1038/s41598-018-36584-w

**Published:** 2019-01-18

**Authors:** Faramarz Djavanroodi, Mahmoud Ebrahimi, Jamal F. Nayfeh

**Affiliations:** 1 Department of Mechanical Engineering, College of Engineering, Prince Mohammad Bin Fahd University, Al Khobar, 31952 Saudi Arabia; 2 Department of Mechanical Engineering, Imperial College London, London, SW7 2AZ UK; 3 Department of Mechanical Engineering, Faculty of Engineering, University of Maragheh, Maragheh, Iran

## Abstract

Tailoring material properties to specific application requirements is one of the major challenges in materials engineering. Grain size is a key factor affecting physical and mechanical properties of polycrystals materials, the presented work enables insight into how the pure nickel properties are affected by application of multi-directional forging (MDF) as a well-known severe plastic deformation method. It is demonstrated that the hardness and wear rate are improved by imposing MDF process. The rate of enhancement is reduced at the higher pass numbers. It is also shown that the application of MDF process changed the mechanism of wear. Non-MDF sample’s surface shows spalling and delamination, while the dominated wear mechanism of final pass sample is peeling with a slight of adhesion. The change of wear mechanism can be associated with the reduction of friction coefficient of the deformed sample. By considering the linear correlation between the hardness and wear rate, a simple and fast procedure is proposed to estimate the wear rate of sample after the different MDF pass numbers using the corresponding hardness magnitude. Additionally, the attained microstructure of the final pass sample shows a combination of ultrafine grains and micro shear bands.

## Introduction

Metals and alloys processed by severe plastic deformation (SPD) have taken up the attraction of researchers due to the requirement of industrial applications for materials with unique properties^1,2^. Accordingly, during the last two decades, various SPD techniques have been introduced and developed for achieving a suitable balance between the favorable properties and cost^3–5^. Through the SPD methods, high magnitude of shear strain is imposed to the specimen causing the increment of dislocations density, formation of low angle grain boundaries, transformation of low to high angle grain boundaries, and generation of ultrafine grain (UFG) materials^6^. Some examples of SPD are including equal channel angular pressing (ECAP)^7^, planar twist extrusion (PTE)^8^, constrained groove pressing (CGP)^9^, equal channel forward extrusion (ECFE)^10^, accumulative roll bonding (ARB)^11^, high pressure torsion (HPT)^12^, friction stir welding/processing (FSW/P)^13^, and multi-directional forging (MDF)^14^.

Although enhancement in mechanical properties of various metals and alloys has been attained via different methods such as grain refinement, further strengthening is extremely favorable. So far, numerous works have been carried out on various properties of post-SPD materials^15,16^. Nanocrystalline titanium with the grain size of 70 nm was fabricated by Shahmir *et al*. using HPT process. This has led to the hardness of 300 Hv and strength of 940 MPa^17^. A comprehensive study was performed on the consolidating pure aluminum powder in tube by equal channel angular pressing and twist extrusion process^18^. The results showed that a nearly full-dense bulk aluminum with an excellent particles’ bonding is fabricated after the first pass of process at 200 °C. Ge *et al*. investigated the feasibility of ultrafine grain ZM21 magnesium alloy as a biodegradable stent, processed by ECAP and low-temperature extrusion^19^. It was found that yield strength of the processed material is improved from 180 to 340 MPa through ECAP process at 150 °C, in addition to good tensile ductility. In the study by Kadkhodaee *et al*., accumulative roll bonding was applied to produce aluminum/silica nanocomposite sheets at room temperature^11^. Their results showed that increasing ARB cycle and silica nanoparticles amount led to the improvement of corrosion resistance in the produced nanocomposite.

Next to the different materials’ properties through the various SPD methods, there is number of studies concentrating on the MDF process of different materials. The study of Zhang *et al*. on the Ti-6Al-4V alloy after three passes of multi-directional isothermal forging showed a homogeneous microstructure with a grain size of about 500 nm, in which yield strength of 1170 MPa, ultimate tensile strength of 1190 MPa, and tensile ductility of 10.4% are attained^20^. Various annealing operations were investigated by Takayama *et al*. on the room-temperature MDF copper processed with the strain of 0.4 to 6.0^21^. Accordingly, conventional discontinuous static recrystallization occurs during the low-strain magnitude. It means that the nucleation of new grains is taking place by long-distance migration of their boundaries, leading to a full-annealing condition. Investigation of Huang and Zhang on the MDF process of AZ31 magnesium alloy up to 36 passes at different temperatures revealed that the best temperature to achieve a specimen with uniform mechanical properties is 350 °C^22^. The microstructural and mechanical properties of Cu-Al alloy was studied before and after the MDF process^23^. The results indicated that conventional dislocation subdivisions, twinning, and shear banding are the main grain refining mechanisms. Also, deformation twinning and shear banding are increased by decreasing stacking fault energy which reduces the final grain size. The study by Miura *et al*. on multi-directionally forged AZ61 magnesium alloy after five passes led to an excellent balance of strength and ductility at the ambient temperature^24^. Additionally, several experiments have been performed on the MDF process of pure aluminum and Al-4wt.% magnesium alloy^25^, Mg-6Al-1Zn alloy^26^, AZ31B magnesium alloy^27^, 2A14 aluminum alloy^28^, and Mg-Gd-Y-Ag-Zr alloy^29^.

It should be mentioned that MDF has a large potential for fabrication of ultrafine grain and even, nanostructure bulk materials which are suitable for industrial applications as compared to the other forging types; hence, it is far attractive to produce large-size metals and alloys with both great strength and good ductility. Also, the most advantages and disadvantages of the MDF process in comparison with the other SPD methods can be listed as follows^30,31^:

- MDF has simple die geometry, high efficiency, and great repeatability^30^.
- MDF can be exerted via classical forging machine with no need to particular devices.
- MDF can be utilized for brittle materials at room temperature due to the application of low strain rate compared to the ECAP and HPT processes^32^.

- Grain refinement ability is less than other SPD methods.
- Grain size reduction is not homogeneous through the sample. This can be compensated by pass number increment^33^.

Although nickel is soft in its pure situation, an extensive variety of applications at chemical processing and electronics has been reported due to its appropriate electrical conductivity, suitable corrosion resistance and low expansion at high temperature. Therefore, it seems that new opportunities and possibilities are opened for industries if the mechanical properties of commercial pure nickel (CP-Ni) is enhanced considerably^24,34,35^. This work has been missioned by considering that there is a little study on the MDF process of CP-Ni. Accordingly, CP-Ni was multi-directionally forged at room temperature up to 6 passes. Then, Vickers hardness measurement, wear behavior, and microstructural observation of the processed sample were performed to investigate and compare with the initial condition.

## Experimental Procedure

The material used for this research is commercial pure nickel with a purity of 99.65 weight percentage (nickel 200). The prepared samples with the dimensions of 15 mm × 15 mm × 12 mm were annealed at 800 °C in an argon atmosphere followed by furnace cooling. This led to the samples with a low hardness, good ductility and an equiaxed grain size of about 34 µm.

**Figure 1:**
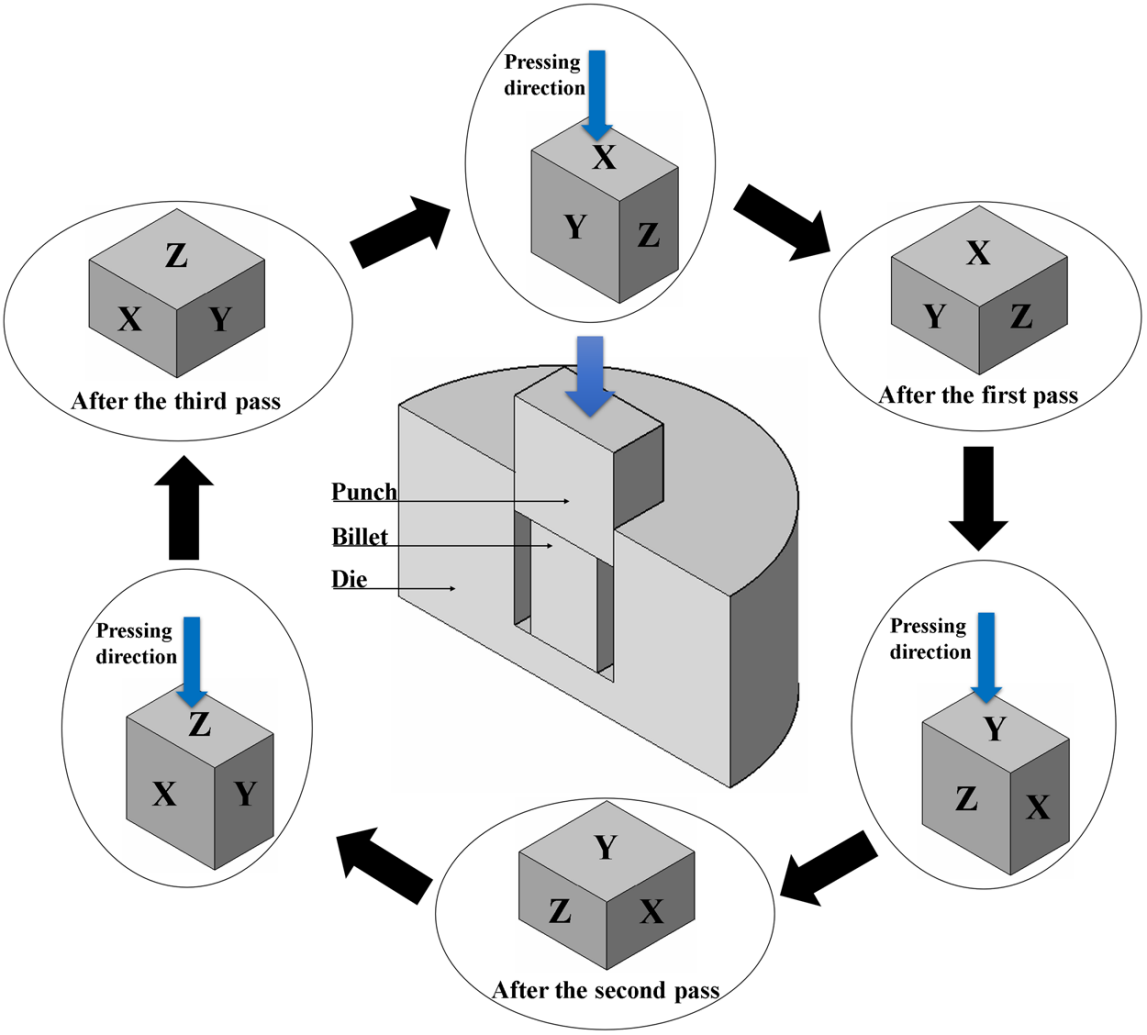
Schematic representation of the MDF process and the sequence of pass number.

MDF process was performed by a mechanical testing machine at the initial strain rate of 3 × 10^−3^ s^−1^. The process was done at room temperature up to six passes with 90° rotation around forging direction between each pass. Figure 1 represents schematically the MDF die set-up and the pass number sequence through the processing operation. As can be observed, this type of MDF die design ensures that each pressing operation is on a different face and three subsequent passes compress the sample in all three directions. It should be noted that molybdenum disulfide paste was utilized in order to reduce frictional force of die-sample-punch interfaces during the operation. The aforementioned processing condition causes 20% reduction in height of the sample during forging. This imposes the effective plastic strain of about 0.26 according to the eq. (1). In this relationship, H and W are the height and width of the deformed sample, respectively^36,37^.

1 $${{\rm{\varepsilon }}}_{{\rm{eq}}}=2/\sqrt{3}\,\mathrm{ln}\,({\rm{H}}/{\rm{W}})$$

Following the MDF process, deformed samples with different pass numbers were cut along the plane perpendicular to the forging axis using a wire-cutting electric discharge machine (EDM). Then, their hardness measurement, wear resistance, and microstructure observation were achieved and compared with the initial condition. In order to remove surface damage due to the EDM operation and prepare samples for the aforementioned tests, they were mechanically ground and polished up to 1 µm. It is important that operation heating does not change the surface properties of the samples during cutting. Micro-hardness test by utilization of Vickers square based pyramid diamond indenter with an included angle of 136° between the opposite faces, was performed on the selected planes of initial and processed nickel samples according to the ASTM E92. The magnitude of the imposed load and dwell time were respectively equal to 200 g and 10 s. sixteen measurements were recorded for each pass, and the average magnitude was reported.

Furthermore, wear behavior of nickel 200 was carried out on the initial and processed conditions with the different pass numbers. This test was done using a linearly reciprocating ball-on-disc sliding method in accordance with the ASTM G133. The wear tests were performed at the ambient temperature with the humidity of about 45% under dry condition. Also, the wear tests were done with a sliding speed of 0.24 m/s, and a total sliding distance of 288 m. Additionally, wear mass loss was obtained by means of a digital balance device by measuring the difference in the weight of the specimen before and after the test. As known, wear rate and friction coefficient are two important factors of surface engineering. Accordingly, their magnitudes were obtained and investigated before and after the MDF process up to the six passes. Wear rate was calculated by dividing the mass loss per traveled distance, see eq. (2). Also, friction coefficient was obtained by dividing the frictional force (F) per applied normal load (N)^9,38–40^. Additionally, the surface morphology of worn samples was investigated using a scanning electron microscopy (SEM) to study wear mechanism of the initial and final pass of pure nickel.

2 $${\rm{Wear}}\,{\rm{rate}}={\rm{Mass}}\,\mathrm{loss}/\mathrm{Traveled}\,{\rm{distance}}$$

3 $${\rm{F}}={\rm{\mu }}{\rm{N}}$$

## Results and Discussion

**Figure 2:**
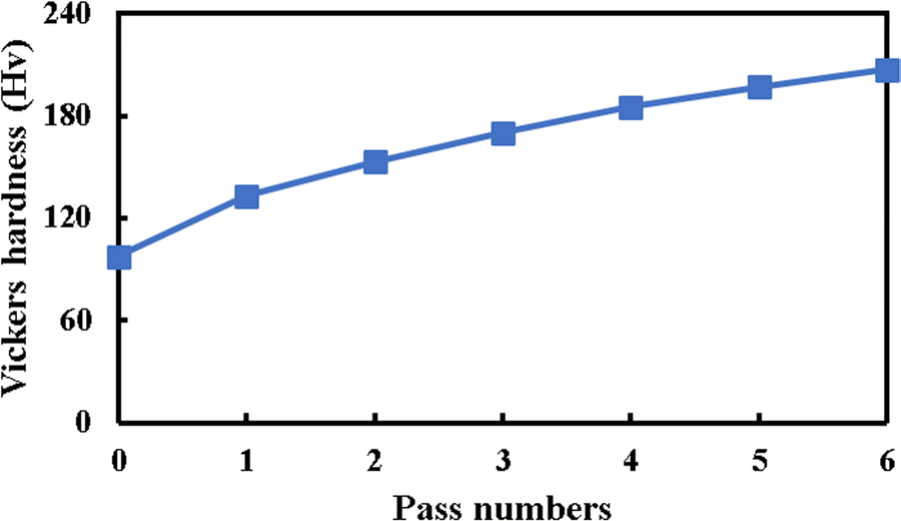
Vickers hardness magnitude of pure nickel before and after the MDF process up to six passes.

Multi-directional forging of nickel 200 results in, the considerable change in hardness behavior. Figure 2 represents the hardness magnitude of nickel sample after the first, fourth and sixth passes of the MDF process accompanied with the initial condition for better comparison. It is clear that the sample shows a sizeable hardness magnitude after the process, and this magnitude is increased by adding the pass number. For instance, about 37%, 74% and 112% improvement at the hardness magnitude are respectively achieved after the first, fourth and sixth passes of the MDF process as compared to the initial condition. Additionally, the increasing trend of hardness behavior is reduced by addition of MDF pass numbers. For example, about 37% improvement of hardness is obtained by applying for the first pass as compared to the initial condition, while it grows only 5% by imposing the sixth pass of the process in comparison with the fifth one. Interestingly, this extraordinary hardness improvement by means of the MDF process is actually beyond what can be attained by the conventional metal forming processes. The significant enhancement at the hardness behavior can be associated with a remarkable refinement of the structure through introducing high density of dislocations. A similar trend of hardness behavior was reported in the ECAP of commercial AZ31 magnesium alloy^41^, ECAP of tungsten heavy alloy^42^, HPT of ZK60A magnesium alloy^43^, and accumulative back extrusion of pure copper^44^.

**Figure 3:**
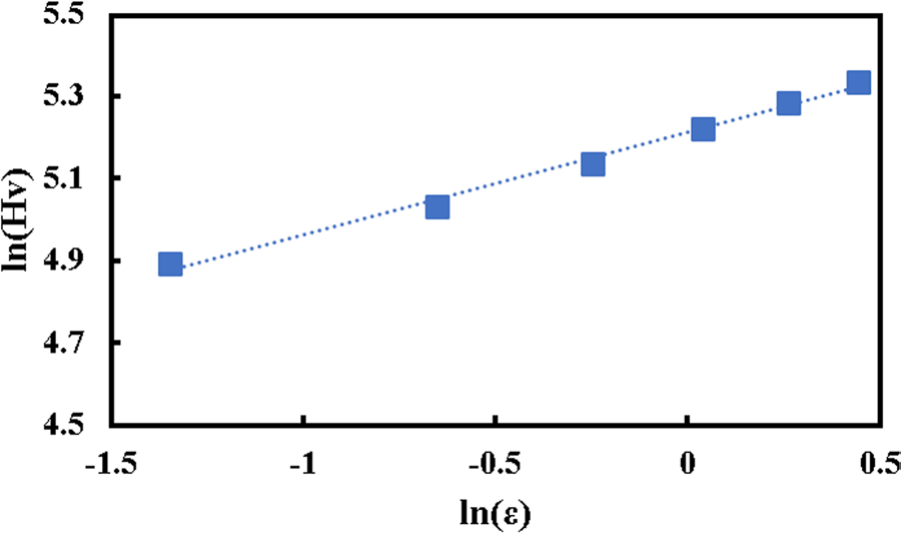
Logarithmic plot of Vickers hardness versus imposed plastic strain during the MDF process of pure nickel.

Quantitative analysis was carried out in order to understand the hardness behavior of pure nickel during the MDF process. Accordingly, hardenability behavior of pure nickel was achieved through the relationship of eq. (4)^43^. In this equation, Hv, ε_eq_, K_H_ and n_H_ represent Vickers hardness, imposed equivalent plastic strain, hardness coefficient, and hardenability exponent, respectively. For this aim, a logarithmic plot of hardness versus equivalent strain was extracted; see Fig. 3. Afterwards, dotted line was fitted to describe a trend of all datum points, in which the R-squared value is equal to 0.9933. This approach leads to the calculation of hardness coefficient and hardenability exponent. The results show that the hardness coefficient is 183.4 Hv, and hardenability exponent is 0.2493. The aforementioned method seems an acceptable approach for quantitative analysis of hardness evaluation of materials through various SPD processes. Previous works reported hardenability exponent of 0.031 and 0.07 are attained for the Ti-6Al-4V and ZK60A after the different condition of HPT process, respectively^43,45^.

4 $${\rm{Hv}}={{\rm{K}}}_{{\rm{H}}}\,{{\rm{\varepsilon }}}_{{\rm{eq}}}^{{{\rm{n}}}_{{\rm{H}}}}$$

**Figure 4:**
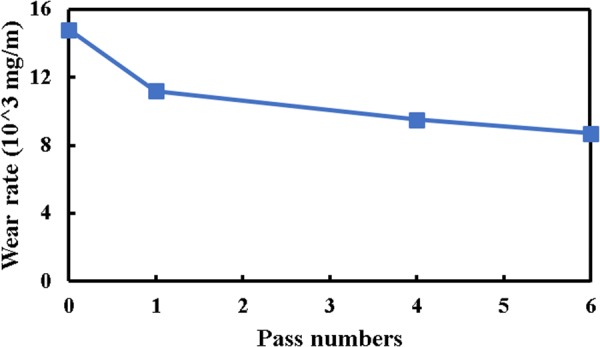
Wear rate of pure nickel before and after the MDF process up to six passes.

The ball-on-disc dry sliding wear test was performed on the pure nickel before and after the MDF process with different pass numbers. As mentioned above, all tests were carried out for a period of 20 min, relating to a sliding distance of 288 m with the speed of 0.24 m/s. Figure 4 represents the wear loss of sample at the various MDF pass numbers. Accordingly, the wear rate of sample is decreased due to the MDF process. The results indicate that the wear rate magnitude of pure nickel is reduced by 27%, 37% and 42% at one, four, and six passes of the MDF process as compared to the initial condition, respectively. Improvement of wear rate by means of MDF process is a prominent factor for choosing material at applications that there is a relative motion between contact surfaces.

**Figure 5:**
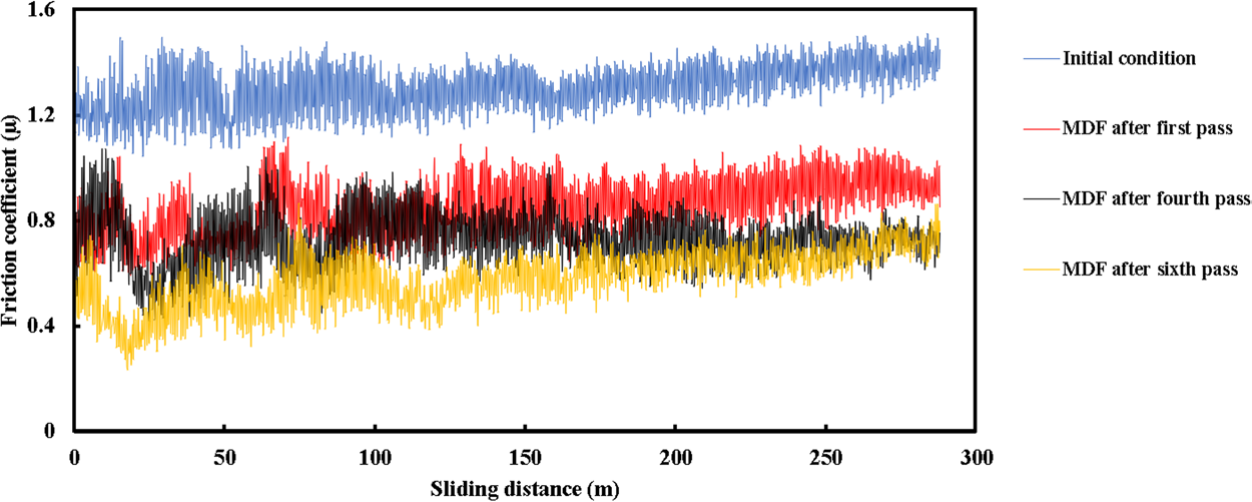
Variation of friction coefficient within the sliding distance of pure nickel before and after the MDF process up to six passes.

Additionally, the friction coefficient is estimated by means of the eq. (3). Accordingly, friction coefficient versus sliding distance of pure nickel under the applied normal load of 20 N was achieved for the various MDF pass numbers. The results are represented in Fig. 5. It is obvious that the friction coefficient grows precipitously at the beginning, thereafter, reaches a steady-state level up to the end. It is found that average friction coefficient of pure nickel which is about 1.307 at the annealed condition, reaches 0.851, 0.731 and 0.579 after the first, fourth, and sixth passes, respectively. There are about 35%, 44% and 56% reductions at the average friction coefficient by utilization of one, four and six passes of the MDF process in comparison with the initial condition. It is worth noting that the magnitude of average friction coefficient is decreased by adding pass numbers, and the decreasing trend is more sizeable after the first pass of the MDF process. The reduction of friction coefficient during the MDF process may be attributed to the increment of hardness magnitude. Moreover, the fluctuations’ amplitude which is wide at first, decreases by addition of the sliding distance. The same trend at the reduction of friction coefficient by imposing intensive plastic strain has been already reported^46,47^.

**Figure 6:**
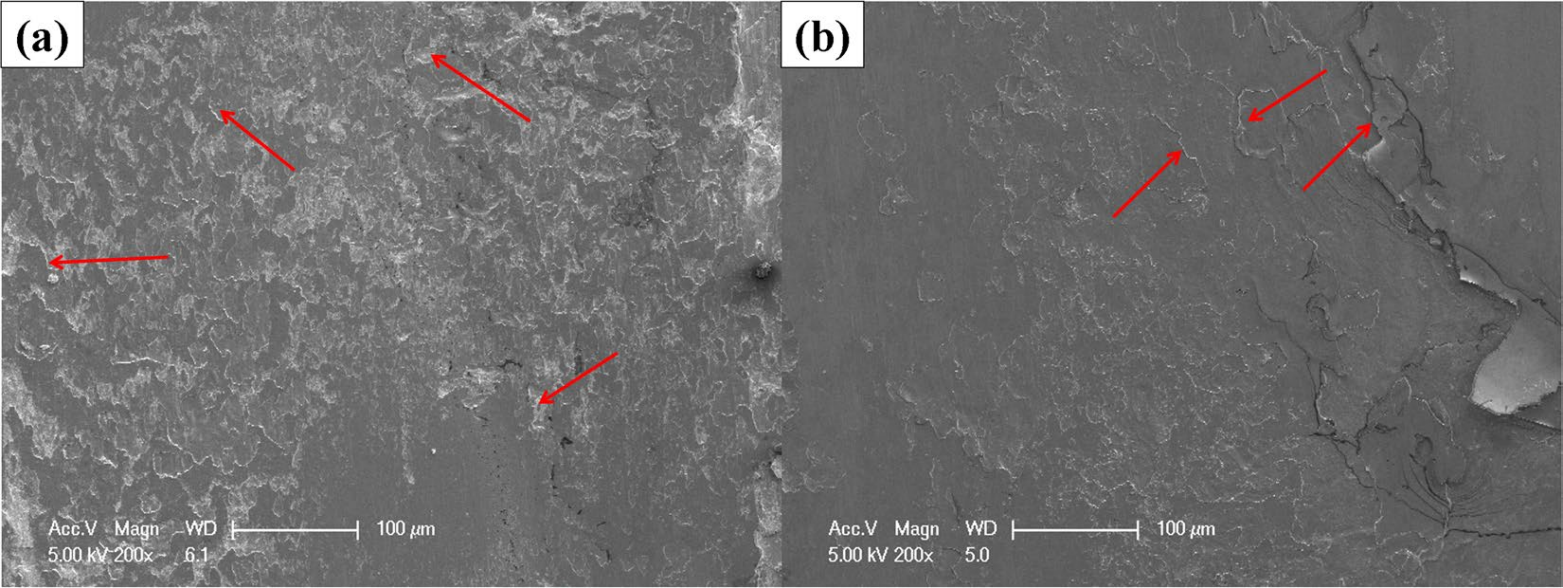
Worn surface morphology of pure nickel (**a**) Before and (**b**) After the six passes of MDF process using SEM micrographs.

Surface morphology of worn sample was analyzed for investigating wear mechanism of pure nickel at initial condition and after undergoing six passes of MDF process. The results are shown in Fig. 6. Accordingly, spalling and delamination are the main wear mechanisms, dominating on the worn surface of the initial sample next to the slight adhesion. Some of the spalling are distinguished by red marks in Fig. 6a. The spalling mechanism is generated due to the crack formation that is perpendicular to the sliding direction. Also, adhesion is created due to the micro joints between the pin and disc. Accordingly, nickel as a softer material is broken and subsequently left during the relative movement of the steel pin. Therefore, small voids appear in the sample. The other important observation is that the adhesion and spalling wear mechanisms may be related to each other. As known, adhesion wear mechanism which causes the formation of micro-voids creates stress concentrated region. Hence, microcracks may be initiated and grown at the small voids, which subsequently lead to the occurrence of spalling and delamination. That is to say, this type of wear mechanism causes the increment of wear rate, as seen above. Applying six passes of the MDF process leads to the change of wear mechanism. As can be observed, peeling and a combination of slight spalling and adhesion are the dominated wear mechanisms of the intensive deformed sample. Deformed pure nickel by MDF process leads to the embrittlement of material. Hence, cracks are generated in the sample which leads to the formation of peeling region. As a consequence, the wear rate is improved and the average friction coefficient is decreased as compared to the initial pure nickel. The intensive peeling of worn surface related to the final pass of the MDF process is a sign of imposing severe plastic deformation due to the increment of hardness magnitude^38,39,46,47^.

The aforementioned findings indicate that an inverse relationship exists between the hardness and wear rate of pure nickel before and after the MDF process. Hence, in order to further clarify the hardness and wear relation for the pure nickel at different MDF pass numbers, a new factor entitling hardness-wear index (HWI) is introduced as eq. (5). The results indicate that the magnitude of HWI increases from 6.58 for the initial condition to 11.89 at the first pass, to 19.49 at the fourth pass, and to 23.81 at the final pass. This means the improvement of the material performance by utilizing the MDF process and increasing pass numbers.

5 $${\rm{HWI}}={{\rm{H}}}_{{\rm{V}}}/{\rm{Wear}}\,{\rm{rate}}$$

**Figure 7:**
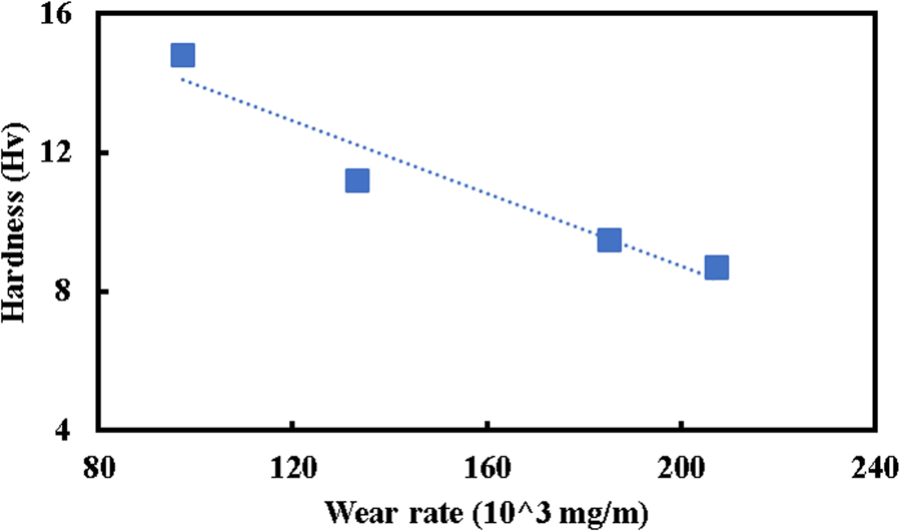
Linear fitting of experimental wear rate and hardness magnitudes of pure nickel before and after the MDF process up to six passes.

It is also worth noting that wear rate determination is a long-term procedure as compared to the hardness measurement. Therefore, it seems obtaining a quick method for estimation of wear rate at different pass numbers (grain size) would be efficacious due to the reduction of both time and cost. Figure 7 represents the obtained datum points for the wear rate and hardness of the first, fourth and final passes of the MDF process. A linear trend line is defined for the plotted data points which shows the acceptable R-squared value (0.9246). Based on the results, it can be said that there is a linear correlation between the hardness and wear rate. The linear relationship also can be a quick way to estimate the wear rate of the pure nickel after the different MDF pass numbers using the corresponding measured hardness magnitude (${\rm{Wear}}\,{\rm{rate}}=-\,17.652{\rm{Hv}}+350.78$).

**Figure 8:**
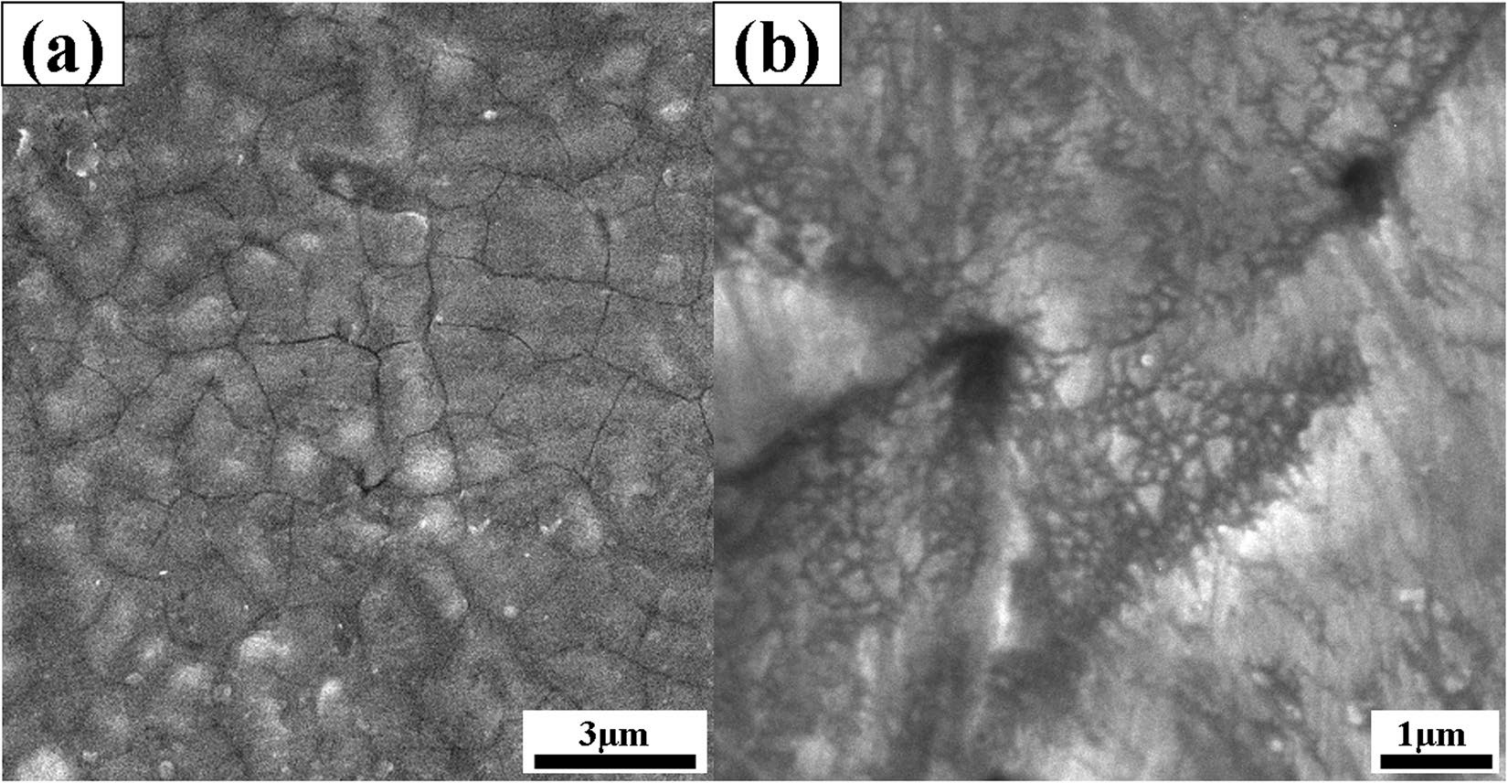
Scanning electron microscopy of pure nickel (**a**) at the initial condition and (**b**) after MDF process up to six passes.

The increment of wear resistance as well as hardness behavior after the MDF process can be associated with the grain refinement of pure nickel. It is generally accepted that the measured hardness of the processed material (H_tot_) can be associated with the simultaneous effect of three parameters according to the eq. (6). In this equation, H_0_, H_sp_, and H_gb_ are the average hardness of the annealed condition, hardness increment due to the second phase, and hardness increment due to the grain refinement, respectively. For pure nickel sample, there is no strengthening effect due to second phase formation. Also, material strengthening due to the grain refinement can be defined according to Hall-Petch relationship (eq. (7)), in which k and d are material constant and average grain size, respectively. To gain a deeper insight into the processed pure nickel, microstructure of the final pass of the MDF process was investigated using SEM, see Fig. 8b. The obtained microstructure appears a combination of ultrafine grains and micro shear bands. It seems that the homogeneous coarse grain structure of initial sample due to the annealing operation is gradually converted to the ultrafine grains through the six passes of MDF process. Micro shear bands which are generated during the initial passes of the process are transformed to the ultrafine grains at the subsequent passes of the process. It is needed to mention that this mechanism which is called continuous dynamic recrystallization (CDRX), is favored at various severe plastic deformation methods^48–51^. Also, the microstructure of the annealed condition consists of equiaxed grains with uniform distribution according to Fig. 8a.

6 $${{\rm{H}}}_{{\rm{tot}}}={{\rm{H}}}_{0}+{{\rm{H}}}_{{\rm{sp}}}+{{\rm{H}}}_{{\rm{gb}}}$$

7 $${{\rm{H}}}_{{\rm{gb}}}=k/\sqrt{{\rm{d}}}$$

It should be considered that significant development can be achieved in the commercialization of UFG/NS nickel produced by MDF process due to its excellent properties, efficient fabrication, and the possibility to fabricate cutting-edge products at the field of food preparation equipment, mobile phones, medical equipment, transport, buildings, and power generation.

## Conclusions

Hardness behavior, wear properties, and microstructural evolution of pure nickel after undergoing six passes of the MDF process were investigated. It is found that the increasing trend of hardness magnitude is reduced by addition of MDF pass numbers. Also, hardenability behavior of sample is predicted using a relationship with the hardness coefficient of 183.4 Hv and hardenability exponent of 0.25. Wear rate of pure nickel is improved by application of MDF process. A maximum of 42% reduction at the wear rate magnitude was observed after six passes of the MDF process in comparison with the annealed condition. Surface morphology comparison of the annealed and final MDF samples showed that the wear mechanisms has transformed from spalling and delamination to peeling and adhesion. A hardness-wear index is proposed as a simple and quick way to estimate the wear rate of the pure nickel after the different MDF pass numbers using the corresponding hardness magnitude. This index is increased from 6.6 at the initial condition to 23.8 at the final pass of MDF process.

## Data Availability

All data generated or analyzed during this study are included in this published article.
